# Pseudobulbar affect as an early manifestation of HIV-related toxoplasmosis

**DOI:** 10.1192/j.eurpsy.2022.743

**Published:** 2022-09-01

**Authors:** N. Arbelo, L. Ilzarbe, M. Gascón González, C. Llach, L. Pintor

**Affiliations:** 1 Hospital Clínic Barcelona, Psychiatry, Barcelona, Spain; 2 Hospital Clínico de Santiago, Servicio De Psiquiatría, Santiago de Compostela, Spain

**Keywords:** HIV, toxoplasmosis, Consultation-Liaison psychiatry, pseudobulbar affect

## Abstract

**Introduction:**

Pseudobulbar affect (PBA) is an emotional disorder characterized by uncontrollable outbursts of laughing and/or crying. It is caused by lesions that damage pathways in the frontal lobe and descending to the brain stem, basis pontis and cerebellum. The main causes are neurodegenerative diseases.

**Objectives:**

To present a case of PBA secondary to cerebral toxoplasmosis.

**Methods:**

The present study is a case report of a patient admitted for HIV-related toxoplasmosis to our hospital. We also researched previous case reports of PBA secondary to CNS infection using a pubmed query.

**Results:**

Mr. JA is a 38-year-old male, with no prior psychiatric or medical history. He reported having had same-sex sexual encounters previously. He was admitted for ataxia and dysarthria in a medical unit, and diagnosed of HIV infection, with a CD4 count of 19 cells/μL. The MRI showed a lesion of 22x19x18mm with ring enhancement predominantly in basis pontis, compatible with toxoplasmosis(Image1). Treatment with sulfadiazine, pyrimethamine and dexamethasone was initiated. After five days of hospitalization he was referred to Consultation-Liaison Psychiatry for involuntary and uncontrollable outbursts of laughing and crying, insomnia, but no other psychopathological symptoms. Therefore, citalopram 20mg per day was started, with reduction on the frequency of outbursts.

**Figure:**
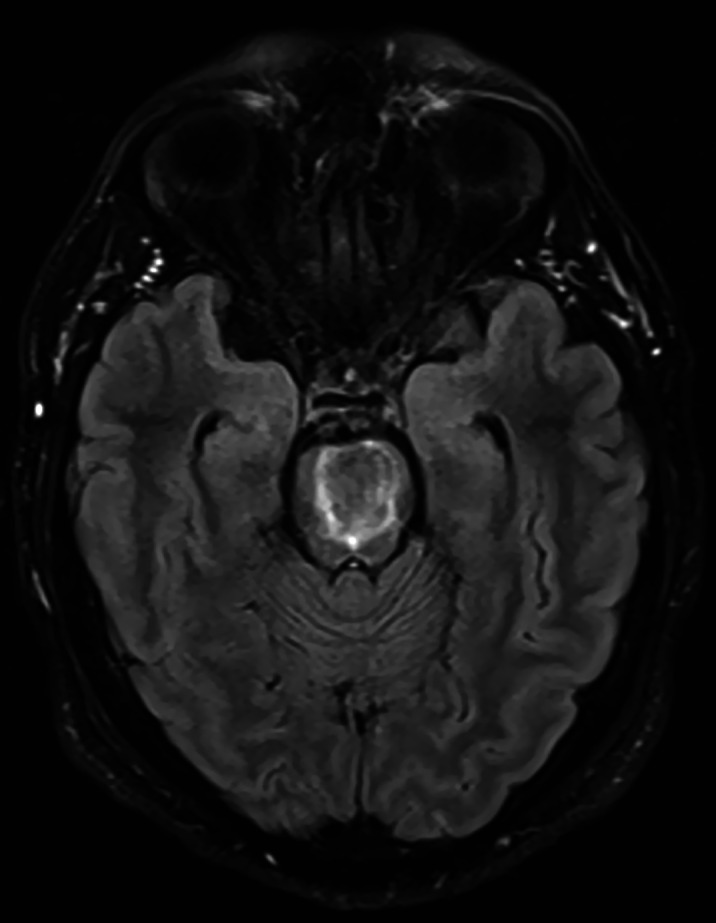

**Conclusions:**

The clinical presentation suggested the diagnosis of PBA due to cerebral toxoplasmosis. Although we found no previous reports of PBA related to HIV infection or toxoplasmosis, the location of the toxoplasmosis lesion is congruent with the typical damaged pathways in PBA. To our knowledge, this is the first report about PBA secondary to HIV-related toxoplasmosis.

**Disclosure:**

No significant relationships.

